# Social determinants of spatial inequalities in COVID-19 outcomes across England: A multiscale geographically weighted regression analysis

**DOI:** 10.1016/j.ssmph.2024.101621

**Published:** 2024-02-07

**Authors:** Esmaeil Khedmati Morasae, Daniel W. Derbyshire, Payam Amini, Tahera Ebrahimi

**Affiliations:** a Research Fellow in Operational Research, Exeter University Business School, University of Exeter, UK; b Department of Public Health and Sports Science, Faculty of Health and Life Science, University of Exeter, UK; c School of Medicine, Keele University, Keele, Staffordshire, UK; d Lecturer in Finance, Business School, Manchester Metropolitan University, UK

**Keywords:** COVID-19, Multiscale geographically weighted regression, United Kingdom, Spatial inequalities, Deprivation

## Abstract

A variety of factors are associated with greater COVID-19 morbidity or mortality, due to how these factors influence exposure to (in the case of morbidity) or severity of (in the case of mortality) COVID-19 infections. We use multiscale geographically weighted regression to study spatial variation in the factors associated with COVID-19 morbidity and mortality rates at the local authority level across England (UK). We investigate the period between March 2020 and March 2021, prior to the rollout of the COVID-19 vaccination program. We consider a variety of factors including demographic (e.g. age, gender, and ethnicity), health (e.g. rates of smoking, obesity, and diabetes), social (e.g. Index of Multiple Deprivation), and economic (e.g. the Gini coefficient and economic complexity index) factors that have previously been found to impact COVID-19 morbidity and mortality. The Index of Multiple Deprivation has a significant impact on COVID-19 cases and deaths in all local authorities, although the effect is the strongest in the south of England. Higher proportions of ethnic minorities are associated with higher levels of COVID-19 mortality, with the strongest effect being found in the west of England. There is again a similar pattern in terms of cases, but strongest in the north of the country. Other factors including age and gender are also found to have significant effects on COVID-19 morbidity and mortality, with differential spatial effects across the country. The results provide insights into how national and local policymakers can take account of localized factors to address spatial health inequalities and address future infectious disease pandemics.

## Highlights

• The drivers of COVID-19 morbidity and mortality vary across England, UK.
• Index of Multiple Deprivation had a significant effect on local COVID-19 deaths.
• Areas with larger ethnic minority populations had higher COVID-19 cases and deaths.
• The effect of both gender and age vary across the country.
• Rates of smoking, obesity and diabetes are less significant at the local level.

## 1 Introduction

Profound spatial disparities in the experience of the COVID-19 pandemic have remained a central focus within the realm of public health research (Albani et al., 2022; Amdaoud et al., 2021; Bourdin & Levratto, 2023; Burlina & Rodríguez-Pose, 2023; Feng, 2023; Gaia & Baboukardos, 2023; Lak et al., 2021; Mansour et al., 2021; McGowan & Bambra, 2022; Morrissey et al., 2021; Shi et al., 2023; Sun et al., 2021; Wang et al., 2023; Welsh et al., 2022). To illustrate, at the outset of the pandemic, the cumulative death rate within the most socioeconomically deprived quintile of local authorities exceeded that in the least deprived quintile by a staggering 54%. This disparity endured throughout the initial nationwide lockdown (Welsh et al., 2022). A similar pattern emerges from analyses of COVID-19 cases in England, with similar findings underscored by studies conducted by Morrissey et al. (2021) and Welsh et al. (2022).

To shed light on these disparities, a cohort of scholars has embraced the syndemic framework as an avenue for explicating the synergistic interplay between health-related attributes at the individual level and the intricate web of contextual factors inherent to particular locales (Albani et al., 2022; McGowan & Bambra, 2022). While the individual factors include aspects such as chronic health conditions (i.e. diabetes and obesity) and age, the location-based aspects correspond to the social determinants of health and compromises of measures of deprivation, unemployment rates, job security, income inequality, healthcare accessibility, etc. (Braveman & Gottlieb, 2014; Paremoer et al., 2021). This intricate interlocking of individual and place-based factors yields a tapestry of diverse arrangements governing exposure, transmission, susceptibility, and vulnerability to the COVID-19 virus. These intricate dynamics converge to precipitate distinct patterns, which ultimately manifest as geographic or spatial imbalances in the trajectory and consequences of the pandemic (McGowan & Bambra, 2022). In this way, existing spatial inequalities in health and social measures engender spatial inequalities in COVID-19 morbidity and mortality.

Alongside this burgeoning body of evidence and scholarly discourse on spatial inequalities during the pandemic within the UK, a recent policy agenda known as “Levelling-Up” has emerged (Fransham et al., 2023). This agenda is designed with the overarching objective of mitigating regional disparities and augmenting the socio-economic standing of local communities. Some scholars posit that this policy initiative is a manifestation of accumulated evidence now finding resonance in political spheres (Davey et al., 2022; Fransham et al., 2023). However, notwithstanding the wealth of insights into inequalities and the concerted drive toward Levelling-Up, a substantial void in knowledge persists regarding the intricate nexus between socioeconomic factors, the social determinants of health and chronic health conditions, and the pandemic experience across diverse spatial strata (Albani et al., 2022; Feng, 2023). To be precise, our comprehension of the occurrence and intensity of these interrelationships across distinct localities in England remains confined, with prevailing studies often assuming a uniform pattern of associations (Greenhalgh et al., 2022; Sun et al., 2021; Welsh et al., 2022). Unraveling whether these relationships demonstrate consistent ubiquity or are localized to specific regions assumes paramount importance. This is pivotal in allowing policymakers to shape precisely targeted policies that proactively address disparities and cultivate resilience against potential future pandemics (Albani et al., 2022).

To bridge this knowledge gap and facilitate evidence-based policymaking, our study employs Multiscale Geographically Weighted Regression (MGWR) (Fotheringham et al., 2017; Wang & Wu, 2020). This analytical approach enables us to investigate spatial dynamics within the impact of socioeconomic factors, the social determinants of health and chronic health conditions on COVID-19 mortality and morbidity across England. Through tailored neighborhood matching and robust regression analyses, we aim to unravel the intricate associations that contribute to spatial inequalities in the pandemic's impact across England.

In the subsequent sections, we provide detailed insights into the previous literature, our general causal assumptions, data sources, explanatory variables, and the MGWR model that underpin our study. Our aspiration is to unearth nuanced insights into the spatial distribution of COVID-19 outcomes, thereby informing targeted policy planning that addresses health and spatial disparities while enhancing societal resilience.

## 2 Literature review

**Fig. 1:**
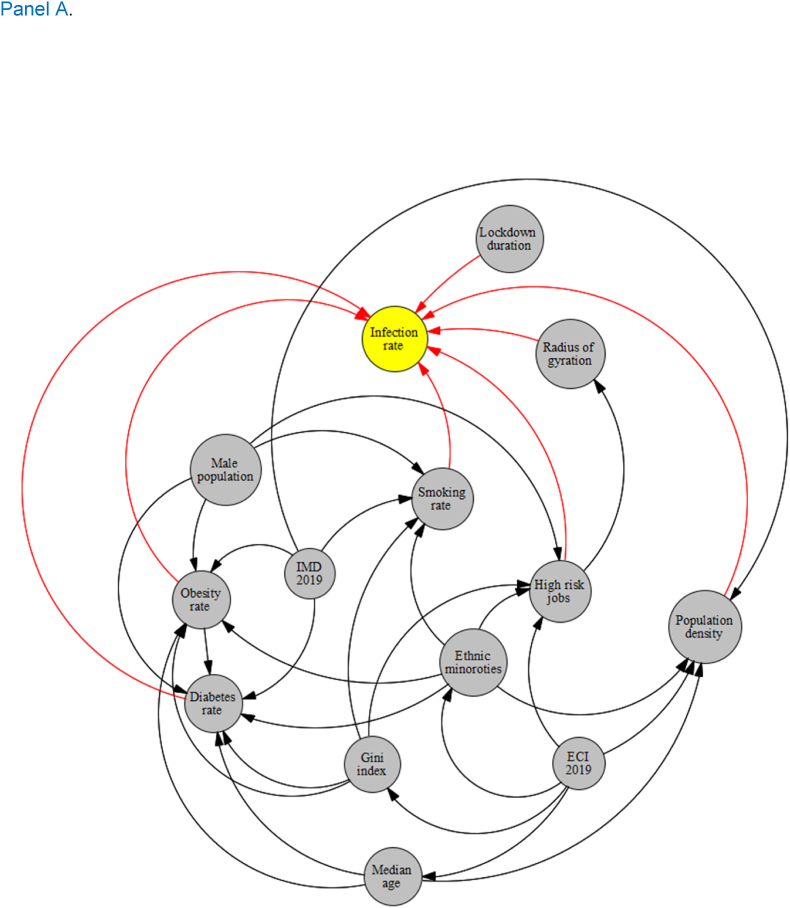

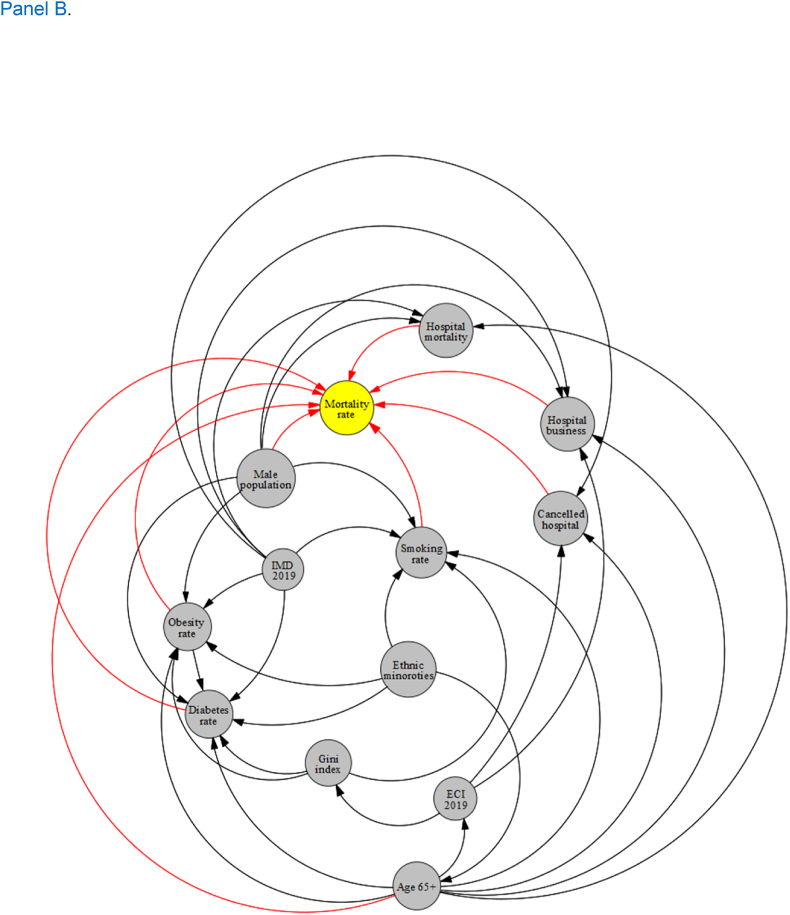
Directed Acyclic Graph (DAG) for COVID-19 morbidity (Panel A) and mortality (Panel B)

Several social and individual factors have been shown to be associated with spatial inequalities in COVID-19 morbidity and mortality in the literature. Namely, there is evidence that the Index of Multiple Deprivation (IMD) has had a significant impact on differences in COVID-19 incidence, hospitalisation, and mortality in the UK localities (McGowan and Bambra, 2022, Morrissey et al., 2021, Upshaw et al., 2021). In a similar vein, there is evidence that income inequality measured by Gini index played a key role in the pandemic outcome at population levels (Burlina & Rodríguez-Pose, 2023; Gaia & Baboukardos, 2023). Further, there is evidence that local economic complexity is a strong predictor of COVID-19 outcomes (Nguyen et al., 2024), above and beyond income and inequalities measures (Vu, 2020). Existing literature has demonstrated that pandemic outcomes were strongly related to population density across localities and regions as higher density facilitated increased exposure and virus transmission (Wong & Li, 2020). There is also strong evidence that higher proportions of ethnic minorities (Sze et al., 2020), men (Zheng et al., 2020), smokers, people who are obese or overweight, people with diabetes, and people over 65 years old (Gao et al., 2020; Hussain et al., 2020) significantly increased the risk of infection, hospitalisation, and death due to COVID-19 in localities. According to some evidence, median age and the age structure of local authorities can also shape their COVID-19 outcomes (Starke et al., 2021). There is also some evidence that people who were working in occupations with a higher chance of human contact (e.g. taxi drivers, guards, food factories, etc.) suffered from higher levels of COVID-19 infection and death, especially in localities with agglomeration of such job owners (Green & Semple, 2023). Moreover, mobility (radius of gyration) was a strong factor in shaping the pattern of infection and mortality in most regions as higher mobility increased the chance of human contact and made people more prone to exposure, infection and death (Santana et al., 2023). Finally, there is evidence that pre-pandemic healthcare preparedness had also significant impacts on the pandemic outcomes in local areas (Gaia and Baboukardos, 2023, Bollyky et al., 2022),

Although the primary analysis of this study was to investigate the association between the above-mentioned factors and COVID-19 outcomes using spatial data analysis (because we assumed each location has a potentially locally dependent mechanism of association), a Directed Acyclic Graph (DAG) approach was primarily used. DAG allows us to explore the general causal pathways that we assumed might link these potential explanatory variables to the outcome variables of COVID-19 morbidity and mortality rates (Steiger et al., 2021; Tennant et al., 2021). We construct separate DAGs for COVID-19 morbidity and mortality, our two outcome variables of interest. The DAGs, constructed based on our literature review, consists of nodes representing variables and directed arrows indicating causal connections. These relationships were meticulously determined to guide the causal interpretation of the arrows, imposing constraints based on the literature review's consensus. In the DAG constructed for morbidity rate, variables such as lockdown duration, radius of gyration, population density, high-risk jobs, smoking rate, obesity rate, and diabetes rate were identified as directly linked to the infection rate. Similarly, variables including hospital mortality, hospital business, canceled hospital appointments, smoking rate, gender (male), obesity rate, diabetes rate, and older age were determined to be directly associated with the mortality rate in the DAG related to mortality. Other variables like ethnicity, IMD, and Gini index served as mediators in the causal graphs (Fig. 1, Panel A and B). This detailed delineation of variables provided a robust framework for subsequent spatial analysis.

## 3 Methods

### 3.1 Spatial unit of analysis

**Fig. 2:**
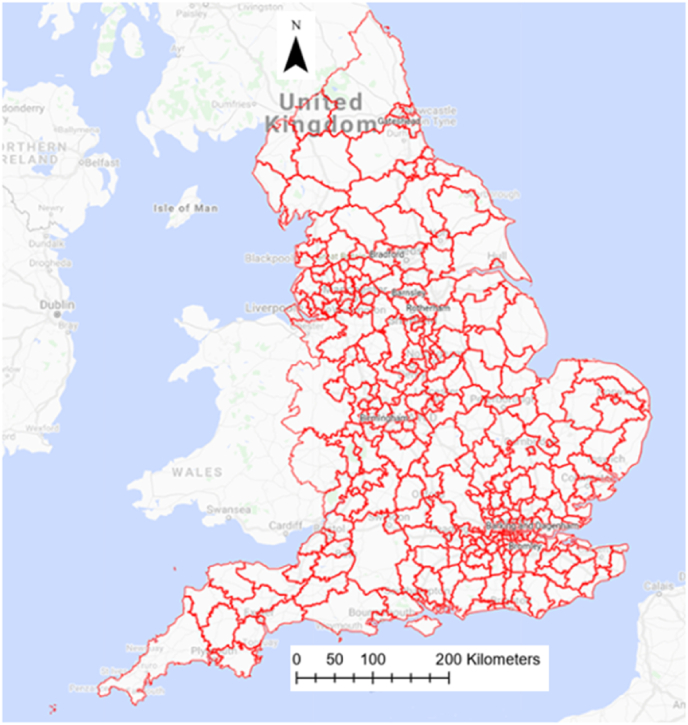
A map of 326 local authorities in England illustrated by red colored delineations. (For interpretation of the references to color in this figure legend, the reader is referred to the Web version of this article.)

The basic unit of analysis in the study was local authority. We used data from 326 local authorities in England (Fig. 2) to conduct the MGWR analyses. The local authorities in England are made up of 5 different types and two unique cases: county councils (Wang & Wu, 2020), district councils (164), unitary authorities (62), metropolitan districts (Steiger et al., 2021), London boroughs (Green & Semple, 2023), City of London (Burlina & Rodríguez-Pose, 2023), and Isles of Silly (Burlina & Rodríguez-Pose, 2023). Local authorities are officially responsible for all the public services and facilities in a particular (geographically delineated) area.

### 3.2 Dependent variables

Our two outcomes used as dependent variables were COVID-19 related morbidity and mortality. In particular, COVID-19 morbidity was measured as the number of confirmed COVID-19 cases per 100,000 members of the population. Similarly, COVID-19 mortality was given as the number of deaths within 28 days of a confirmed COVID-19 diagnosis per 100,000 members of the population.

### 3.3 Explanatory variables

The process for choosing the relevant explanatory variables that could explain spatial inequalities in COVID-19 experience across local authorities is described above. The included variables that emerged from the literature review and DAG are now outlined. Firstly, measures of IMD and the Gini coefficient were included as explanatory variables. Moreover, we used a recently emerging approach, called economic complexity, in the economic literature that captures the productive capacity and economic structure of local economies through an analysis of their products and industries. We include that as an index of the structure of local economies that might better capture the nuances of local-level economic status, enriching the insight that the Gini index can bring to the study. We measure population density as the number of people per square kilometer in each locality. We also included the proportion of men, ethnic minorities, people over 65, and the median age, to account for demographic differences between local authorities. We further included the proportion of people who smoke, the proportion with diabetes, and the proportion who are overweight or obese, to further account for pertinent population differences between local authorities. We also include the proportion of people working in higher-risk occupations. The mobility (gyrus of radiation) measure showed the average change in the distance people moved away from their house one year before and one year after the pandemic started in each local authority. As a proxy for healthcare system preparedness, we added the following three variables; hospital business, hospital cancellation, and hospital mortality. Hospital business was defined as the percentage of adult critical care beds occupied in each locality. Hospital mortality was defined as the rate of death (out of total death rate) that happened in a hospital setting in each locality and given by the Summary Hospital-Level Mortality Indicator (SHMI). Hospital cancellation was defined as the average number of urgent operations cancelled in the healthcare facilities in a given locality. A detailed description of the explanatory variables and their measurement procedure is provided in Appendix A.

### 3.4 Data sources

The required data for analyses was obtained from several UK official sources. COVID-19 mortality and morbidity rates (per 100,000 population) data was obtained from the government's COVID-19 dashboard that updates the morbidity and mortality rates data for each local authority on a daily basis since shortly after the first case in the UK was reported (COVID-dashboard-UK, 2021). We collected the COVID-19 data from 1st March 2020 until 1st March 2021, covering a period from the first wave and lockdown to the third wave and lockdown, just before the roll-out of the vaccine program. Our lockdown duration variable is derived from the Health Protection (Coronavirus, Restrictions) (England) Regulations 2020 and the Health Protection (Coronavirus, Restrictions) acts of parliament which defined the lockdown restrictions. Public health data regarding obesity, diabetes diagnosis, smoking, hospital business, hospital mortality, and hospital cancellation rates were obtained from Public Health England and National Health Services (NHS) data repositories (Public-Health-England, 2021). Data regarding population density, IMD, and proportion (%) of ethnic population, male population, and people in high-risk occupations were obtained from the Office for National Statistics (ONS), which provides disaggregated demographic and economic data for all local authorities across the country (nomis). Most of the above mentioned data related to 2020 and 2021, when pandemic had the grip on the country and when our morbidity and mortality data relates to. However, IMD data related to 2019 when the index was last updated (GOV, 2019). The UK atlas of inequalities was used to obtain the Gini index data for 2019 (Rae AN, 2019). We used data from ONS to calculate the proportion of people who used to work in such occupations over the pandemic time in each locality. Change in mobility (or gyrus of radiation) data (pre- and post-pandemic) was collected from anonymous mobile phone users who opted-in to give access to their location data anonymously. The mobility data was anonymized and the data provider (cuebiq) applied noise to sensitive areas, such as home locations, to prevent re-identification. Finally, industrial employment data from the Business Register and Employment Survey for the year 2019 was used to measure local economic complexity index (BRES, 2019).

### 3.5 Multiscale Geographically Weighted Regression (MGWR)

To describe the continuous and categorical variables, we used mean (standard deviation) and frequency (percentage) respectively. To do the statistical analysis, we utilized MGWR. This spatial regression technique allows for the investigation of spatially varying relationships between a dependent variable and multiple independent variables, accounting for potential spatial non-stationarity.

MGWR is an extension of geographically weighted regression (GWR) designed to avoid misspecification of one or more of the scales when multiple distinct spatial scales generate data and GWR is applied, which can lead to parameter estimates that are biased. To address this issue, a more realistic assumption is that each relationship may occur at a different scale. To achieve this, MGWR extends GWR by recasting it as a generalized additive model, allowing for each variable to be associated with a distinct bandwidth – which represents the size of the local unit for each regression analysis (e.g. the number of local authorities). MGWR therefore represents a generalized and more flexible version of GWR. Let the location $\left(u_{i},v_{i}\right)$ where $u_{i}$ and $v_{i}$ represent the spatial coordinates (longitude and latitude) associated with the midpoint center of a given local authority (i). Further, $X_{ij}$ is a matrix of m explanatory variable for each of i local authorities, and $\beta_{bwj}\left(u_{i},v_{i}\right)$ is the jth coefficient based on the bandwidth selected (bw) for a given calibration at location i. Finally, $\varepsilon_{i}$ is the error term and $y_{i}$ is the dependent variable. The model formula can then be written as follows:

$$y_{i}=\sum_{j=1}^{m}{\beta_{bwj}\left(u_{i},v_{i}\right)X}_{ij}+\varepsilon_{i}$$

To implement the MGWR analysis, the data was first organized into a spatial format. The optimal spatial scale was then determined using adaptive kernel bandwidth selection, and a weighted least squares regression model was used to estimate the parameters of the MGWR model. The kernel function, which specifies the degree of influence that each observation has on the estimation of the local model parameters, was selected based on its suitability for the data. Interpretation of the results involved examining the local coefficients of determination (R^2^) and the local parameter estimates. The local R^2^ values provided insights into the degree of spatial non-stationarity in the relationships between the variables, while the local parameter estimates allowed for the identification of regions with significant relationships (Fotheringham et al., 2017; Wang & Wu, 2020). The produced MGWR output then displays and visually represents the estimated effect size of the explanatory variables on the outcomes for each geographical location. This includes both the direction and magnitude of the effects. Here, a positive estimated coefficient indicates a direct, positive impact of the predictor on the outcome. Conversely, a negative coefficient signifies an inverse relationship between the explanatory variable and the outcome.

We also confirmed the presence of spatial autocorrelation in COVID-19 outcomes (using Moran's test) and the suitability of using MGWR. We also tested and ruled out multi-collinearity (using variance influence factors (VIFs)) issue alongside the MGWR to make sure that there was no problem with our explanatory and outcome variables that could bias the MGWR outputs. More information regarding the testing for these issues is provided in Appendix B.

## 4 Results

Table 1 provides descriptive information about explanatory and outcome variables. As it can be seen, on average, there has been 5884.1 (SD = 2056.6) and 188.4 (SD = 63.4) COVID-19 cases and deaths in each local authority in England, respectively. Moreover, people moved fewer miles (−9.7 (SD = 10.6)) than the year before COVID-19 and 61% (SD = 9.23) of the population were obese or overweight. Local authorities, on average, spent 201.38 days (SD = 10.29) in lockdown. The median age was 41 years old and 11.15% of the population were from an ethnic minority background. The average percentage of male population was 48.1%. Further, 61.6% of the population were obese or overweight, 7% were diagnosed with diabetes and 12.3% smoked, the average level of IMD was 19.4 and average Gini coefficient was 0.33. The full spatial distributions of our explanatory variables can be found in Appendix C.

**Table 1:** Descriptive information about explanatory and dependent variables of the study.

Variables	Mean/Percentage	Standard deviation	Minimum	Maximum
COVID-19 cases rate (per 100,000)	5884.1	2056.6	1383.9	11463.7
Covid-19 deaths rate (per 100,000)	188.4	63.4	50	397.7
Smoking over 18 (%)	12.3	3.9	3.2	27.7
Diabetes rate (%)	8.6	2.1	6.5	10.7
IMD 2019	19.4	7.8	5.5	45
Gini index	0.3	0.02	0.3	0.4
Cancelled hospital (%)	2	3.3	0	25.7
Hospital business (%)	0.8	0.1	0.2	1
Hospital mortality (%)	1	0.1	0.4	1.2
Obesity rate (%)	61.6	9.2	20.6	76.3
Population density (per Sq. Km)	1774.6	2644.6	25.1	16790.7
Age 65 (%)	16.8	4.3	3.7	28.6
High risk occupation (%)	23.1	7.3	6.4	44.6
Change in radius of gyration (Mile)	−9.7	10.6	−62.3	11.2
Median age	41.1	6.5	13.4	54.7
Ethnic groups (%)	11.1	12.8	0	71
ECI 2019	0.05	1	−1.2	4.1
Male population (%)	48.1	5	47.5	53.6
Lockdown duration	201.4	10.3	179	214

**Fig. 3:**
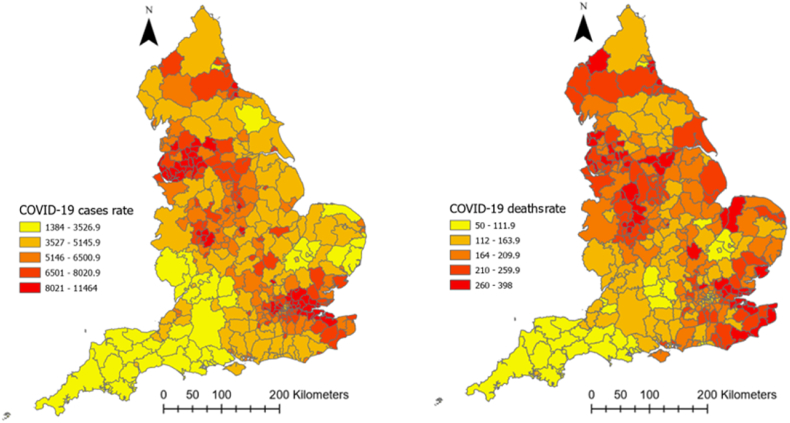
Geographical distribution of COVID-19 morbidity and mortality rates across local authorities in England (the legends show the number of cases/deaths in a color-coded format). (For interpretation of the references to color in this figure legend, the reader is referred to the Web version of this article.)

Fig. 3 illustrates the spatial distribution of COVID-19 cases and deaths rates across the local authorities in England. As can be seen, there was higher morbidity of COVID-19 concentrated in the northwest and southeast of the UK. A similar pattern can be observed for death rate as well, with a higher concentration of mortality in the north, midland, and southeast regions of the country.

**Fig. 4:**
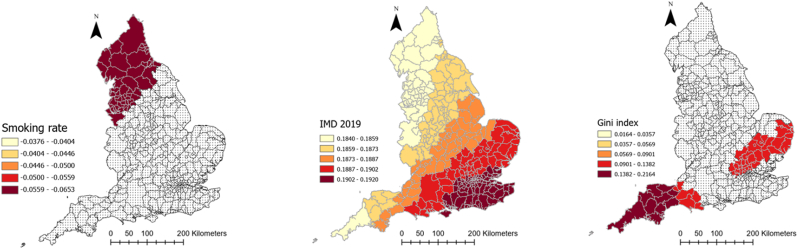

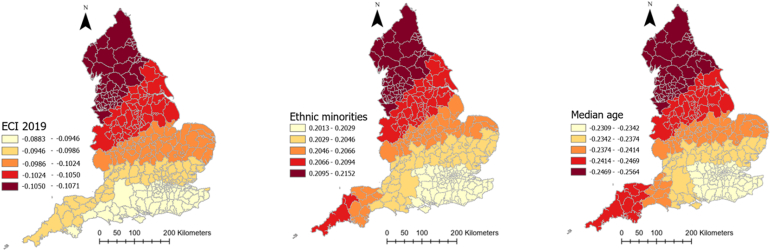

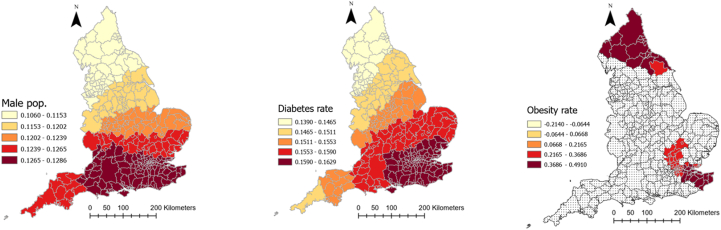

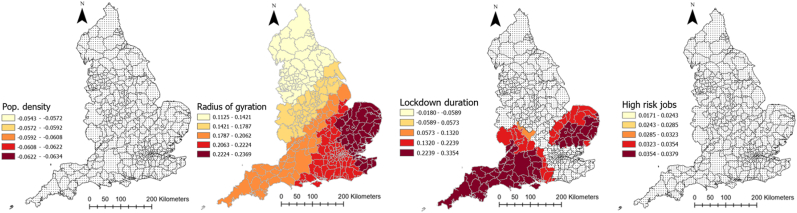
Coefficients of the relationship between COVID-19 morbidity and explanatory variables derived from MGWR analysis

Table 2 represents the outputs of an MGWR analysis for the COVID-19 morbidity rate and its explanatory analyses. The table shows the mean, standard deviation (SD), minimum, maximum, and median of coefficients between COVID-19 and each explanatory variable across all local authorities in England. The last column also shows the percentage of localities for which the corresponding coefficient for each explanatory variable was significant. Fig. 4 shows how these coefficients vary across the country and for which local authorities they are significant. As can be seen, the relationship between population density and the percentage of people with risky occupations with COVID-19 morbidity rate was insignificant in all localities across England. However, the relationship between median age, ECI, and smoking rate with COVID-19 morbidity rate was negative, with different patterns of significance across the localities. To be precise, the negative relationship between median age and the infection rate was significant across all the localities, meaning that in local authorities where people were older, the rate of COVID-19 cases per 100,000 population was lower (mean = −0.203, SD = 0.004). Fig. 4 shows that this relationship was the strongest in the north of the country. Similarly, ECI had a significant negative relationship with COVID-19 cases across all localities, meaning that in places with lower economic complexity, the rate of COVID-19 cases was higher. This relationship gets stronger as one moved north (Fig. 4). Finally, the negative relationship between the smoking rate and infection rate was only significant in 16 percent of localities, all in the country's north.

**Table 2:** Relationship between explanatory variables and COVID-19 morbidity rate in England according to MGWR outputs.

Explanatory variables	Mean	Standard deviation	Minimum	Median	Maximum	Significance (% of localities)
Smoking over 18 (%)	−0.0466	0.0075	−0.0653	−0.0446	−0.0377	54 (16.56)
Population density (People per Sq Km)	−0.0612	0.0022	−0.0635	−0.0620	−0.0544	0 (0)
ECI 2019	−0.1008	0.0050	−0.1071	−0.1024	−0.0883	326 (100)
Median age	−0.2394	0.0065	−0.2565	−0.2379	−0.2310	326 (100)
Diabetes rate (%)	0.1553	0.0059	0.139	0.1567	0.1629	326 (100)
IMD 2019	0.1884	0.0021	0.184	0.1884	0.1921	326 (100)
Gini index	0.0802	0.0468	0.0164	0.1083	0.2164	63 (19.33)
Obesity rate (%)	0.1478	0.1721	−0.2141	0.1380	0.4911	80 (24.54)
Ethnic groups (%)	0.2054	0.0033	0.2014	0.2046	0.2152	326 (100)
Male population (%)	0.1227	0.0058	0.1061	0.1250	0.1286	326 (100)
High risk occupations (%)	0.0312	0.0056	0.0172	0.0329	0.0379	0 (0)
Radius of gyration change	0.1878	0.0384	0.1126	0.2043	0.2369	326 (100)
Lockdown duration	0.1012	0.1151	−0.0590	0.0869	0.3355	100 (30.67)
Intercept	0.11	0.372	−0.784	0.127	1.008	143 (43.87)

Percentage of ethnic groups, diabetes rate, IMD, male population, and radius of gyration in each local authority, on the other hand, had a positive significant relationship with COVID-19 morbidity rate (mean = 0.323, SD = 0.18) across all localities, meaning that having a higher deprivation level, mobility level, percentage of ethnic groups, and male population was associated with higher rates of COVID-19 cases. As Fig. 4 illustrates, for IMD, radius of gyration, and diabetes rate, the relationship was the strongest in the southeast of England, but for ethnic minorities it was the strongest in the north of the country.

Obesity rate, Gini index, and lockdown duration had also a positive relationship with COVID-19, but only in 24, 19, and 31% of local authorities, respectively. For the Gini index and lockdown duration, the relationship was the strongest in the southwest, and for the obesity rate it was the strongest in the north and southeast (Fig. 4).

**Fig. 5:**
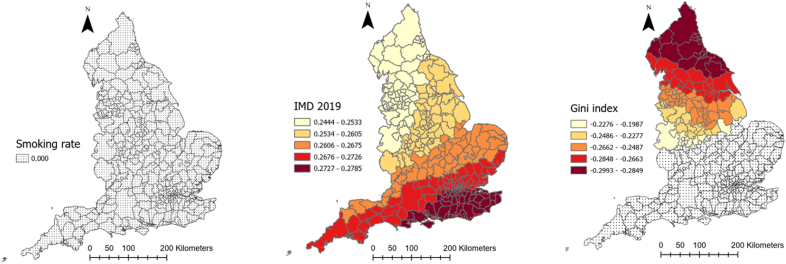

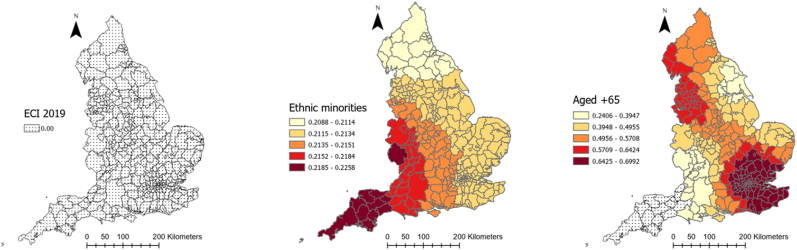

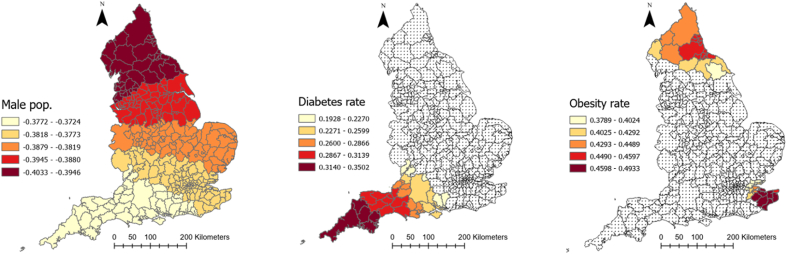

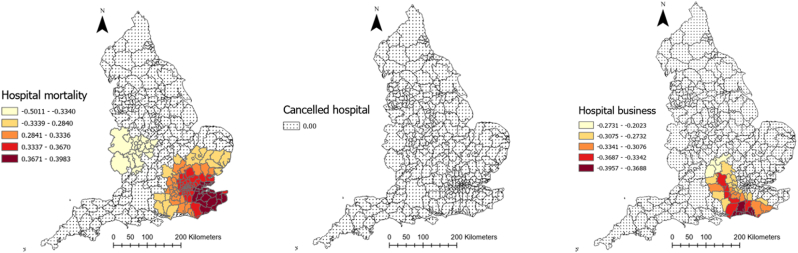
Coefficients of relationship between COVID-19 mortality and explanatory variables derived from MGWR analysis

Table 3 represents the outputs of a MGWR analysis for COVID-19 mortality rate. Fig. 5 shows how these coefficients vary across England and which local authorities they are significant. As can be seen, the relationship between smoking rate, cancelled hospital appointments, and COVID-19 mortality was insignificant in all localities across England. Percentage of ethnic minorities and IMD in each local authority, by contrast, had a positive relationship with COVID-19 mortality rate and this relationship was significant in all local authorities across England. As Fig. 5 illustrates, these two relationships were the strongest in the south (IMD score) and southwest (ethnic minority) of the country. Similarly, the percentage of people aged over 65 in local authorities was also positively associated with COVID-19 mortality, and this relationship was significant in 97% of localities. The relationship was very strong in the southeast of England (Fig. 5) and was insignificant in the southwest.

**Table 3:** Relationship between explanatory variables and COVID-19 deaths rate in England according to MGWR outputs.

Explanatory variables	Mean	Standard deviation	Minimum	Median	Maximum	Significance (% of localities)
Gini index	−0.048	0.17	−0.299	0.056	0.118	118 (37.22)
Cancelled hospital appointments (%)	−0.005	0.005	−0.013	−0.005	0.011	0 (0)
Hospital business (%)	−0.094	0.15	−0.395	−0.067	0.126	44 (13.88)
Male population (%)	−0.383	0.007	−0.403	−0.381	−0.372	317 (100)
Smoking over 18 (%)	0.005	0.006	−0.003	0.004	0.022	0 (0)
Diabetes rate (%)	0.146	0.058	0.088	0.136	0.35	45 (14.19)
IMD 2019	0.263	0.009	0.244	0.265	0.278	317 (100)
Hospital mortality (%)	0.156	0.25	−0.501	0.259	0.398	143 (45.11)
Obesity rate (%)	0.197	0.154	−0.475	0.193	0.493	35 (11.04)
Aged 65+ (%)	0.535	0.141	0.084	0.561	0.699	309 (97.47)
ECI 2019	0.031	0.007	0.02	0.03	0.05	0 (0)
Ethnic groups (%)	0.213	0.002	0.208	0.213	0.225	317 (100)
Intercept	0.072	0.522	−1.335	0.103	1.105	141 (44.47)

Diabetes rate, hospital mortality, and obesity rate were also positively associated with COVID-19 mortality, but the association was significant in less than 50 percent of local authorities. Diabetes rate was only significant in 14 percent of local authorities located in the southwest of the country. Obesity rate was only significant in 11 percent of local authorities, mainly located in the north. Hospital mortality was significant in 45 percent of local authorities, mainly located in southeast (see Fig. 5).

The relationship between percentage of male population and COVID-19 mortality rate was significant across all localities of the country, but the relationship was negative. Moreover, as Fig. 5 shows, the relationship was the strongest in the north. Gini index and hospital business had also a negative association with COVID-19 death rate. But the relationship was only significant in 37% and 14% of local authorities for Gini index and hospital business, respectively. These local authorities are located in the north in the case of Gini index and in the south for hospital business (Fig. 5).

## 5 Discussion

The study used multiscale geographically weighted regression (MGWR) to investigate spatial inequalities in COVID-19 morbidity and mortality rates across local authorities in England. We found higher concentrations of COVID-19 cases and deaths in the northwest and southeast regions. We also found a negative association between median age, economic complexity, and COVID-19 morbidity rate across all local authorities, with the strongest associations in the north. A positive relationship was observed between the percentage of ethnic minorities, diabetes rate, IMD, male population, mobility, and COVID-19 cases in all local authorities, with the strongest relationship in the southeast and north of the country. The lockdown period was also positively related to the infection rate, namely in 30% of localities located in the southwest. Shifting our focus to COVID-19 mortality, the percentage of ethnic minorities and IMD score showed significant positive associations with COVID-19 mortality rate across all local authorities, with the strongest relationships observed in the south and southwest regions. The percentage of people aged over 65 also had a positive significant relationship with COVID-19 mortality, mainly in the southeast region. Further, we found that the effect of population density, and percent of people with risky occupation on COVID-19 morbidity were insignificant across all of England. Similarly, in terms of COVID-19 mortality, economic complexity, cancelled hospital appointments (prior to COVID-19) and the rate of smoking were insignificant across all local authorities in England. These findings underscore the intricate spatial dynamics of COVID-19 outcomes in England, providing valuable insights for targeted interventions and policy formulation. By employing a sequential approach, where the DAG guided the selection of variables and the MGWR illuminated their spatial relationships, the study offers a comprehensive understanding of the complex interplay between explanatory and outcome variables. The integration of these methodologies not only reinforces the causal inferences drawn but also provides a nuanced view of how these relationships manifest across different spatial contexts. The findings from this analytical journey pave the way for targeted interventions and informed policy-making, addressing the multifaceted nature of infection and mortality rates with spatial and causal precision.

Consistent with previous studies highlighting a heightened infection rate among the younger population (Goldstein et al., 2020; Monod et al., 2021), our investigation similarly reveals a discernible inverse connection between median age and COVID-19 morbidity in England. There have been some speculations to explain such a trend among the younger population (except for children who were relatively less susceptible to COVID-19). In particular, it has been suggested that younger members of the population exhibited differing patterns of interactions that led to a higher infection rate among themselves, but avoided transmission to other (i.e. older) groups. In this sense, it may be that younger people showed heightened caution in their interactions with older people and conscientiously avoided the transmission of infection to their older relatives. Also, the older people exhibited an increased sense of prudence, diligently practicing social distancing and limiting social interactions, thereby effectively diminishing their exposure risk compared to younger people (Goldstein et al., 2020; Monod et al., 2021). Moreover, younger adults were the people who needed to work in order to support themselves and their families, therefore having a higher chance to develop the infection (Monod et al., 2021). This observed negative association held true across all local authorities in England, with the relationship strengthening as one moved from the eastern to the western and northern reaches of the country. Notably, the southwest (i.e. Cornwall and Devon counties) of the country exhibited a strong relationship, suggesting the potential influence of regional factors, extra to the abovementioned factors, on this association. Both Cornwall and Devon have relatively high average ages and are more rural compared to other parts of the country. These factors may have contributed to the observed COVID-19 infection patterns in this region, as it provides older individuals with a more conducive environment for effective self-isolation. Interestingly, the relationship between the proportion of people aged over 65 and COVID-19 mortality rate was significant everywhere in the country except for the southwest. This is in line with the pattern of COVID-19 cases as older people had a relatively lower infection rate in this part of the country and their death rate was, as a consequence, lower, in part due to these regional factors (Zheng et al., 2020). We say in part due to regional factors since hospital care features were not significantly associated with the mortality rate in this region of the country. Even the fact that the relationship between the local diabetes rate and COVID-19 mortality is only significant in this part of the country could not influence the negative association between age and mortality in this region, despite increased diabetes prevalence with age. Another issue that might be of interest in understanding the patterns of COVID-19 mortality and morbidity in this region is that the lockdown period was relatively shorter in this region and one of the few places where there was a significant positive relationship between infection rate and lockdown period. Considering all the points mentioned, we can postulate that, due to the geographical features, there is a chance that people in this region of the country got less adherent to the lockdown rules as time passed and were less attentive to physical distancing regulation and this led to higher cases among them, especially among the young (Wright et al., 2022). All these however call for future research to investigate this issue in detail.

A geographically ubiquitous positive significant relationship, stronger in the southwest of the country, was found between the proportion of people from an ethnic minority background in each local authority and COVID-19 death rate. This finding is in line with the accumulated evidence that ethnic minorities were disproportionately affected by the pandemic, both in terms of infection and death rate (Gaia & Baboukardos, 2023; Irizar et al., 2023). Irizar et al. used a systematic evidence mapping method to identify the pathways that led to disproportionate burden of the pandemic on ethnic minorities. According to their mapping, comorbidities, socioeconomic inequalities, neighborhood infrastructure, occupational risks, barriers to healthcare, and COVID-policy effects were the main conditions that led to disproportionate burden of the pandemic among ethnic minorities in UK (Irizar et al., 2023). As Iriza et al. explain, by adopting the syndemic framework, socioeconomic inequalities (i.e. socioeconomic status, deprivation, and employment status) and occupational risk (e.g., healthcare worker status, keyworker status, shift work) led to differential exposure to the virus among ethnic minorities. Further, comorbidities (e.g. diabetes, hypertension, etc.), neighborhood level factors (air pollution, overcrowded housing, housing quality, and ethnic density), and health behaviours (e.g., smoking status, alcohol use, physical activity, diet) led to differential vulnerability to infection and disease among minorities. Additionally, barriers to healthcare (e.g., access to services, culturally sensitive language, and fear of inequitable treatment) led to differential disease consequences. Finally, the differential adequacy of COVID-19 control measures and policies led to the differential effectiveness of control measures to the disfavor of the ethnic people. Configurations of these pathways then led to the observed unequal patterns of the pandemic outcomes among ethnic minorities in the UK (Irizar et al., 2023). Our findings do provide support for some of these pathways, as shown in our DAG for mortality rate. Namely, our findings sit well with the neighborhood-level factors as we show that the proportion of ethnic minorities is significantly associated with the pandemic outcomes across England. But there is little local evidence from the southwest of England to allow us to explore the pathways in this region. There is, however, some local evidence from public health authorities that might help to explain why a stronger relationship between ethnicity and COVID-19 outcomes was found in this region. Namely, some evidence shows that ethnic minorities were up to twice as likely to die from COVID-19 compared with the ethnic majority in this region (OHID, 2021). Also, less than 50% of ethnic minorities do engage in physical activities and up to 30% did not opt for full COVID-19 vaccination (OHID, 2021), although our analysis predates the vaccine rollout. This information, although very incomplete and in need of future research, can somehow help us get a sense of the reasons behind such a strong association observed. Furthermore, all these happen while the proportion of ethnic minorities in the southwest region is significantly lower than the country's average (OHID, 2021). This fact might also help us to explain some of the patterns identified as it seems that due to a lower proportion of ethnic minorities in society, most of the entrenched unequal social structures are still not challenged or re-thought.

The significant positive relationship between IMD and COVID-19 mortality was also present in all local authorities, with the strongest relationship in the southeast. There is a relatively similar pattern in terms of cases. The positive relationship between deprivation and COVID-19 deaths is consistent with previous literature, as shown in our DAGs (Albani et al., 2022; Gaia & Baboukardos, 2023; McGowan & Bambra, 2022; Morrissey et al., 2021). This relationship may be due to the association between higher levels of deprivation and other co-morbidities that may lead to more severe symptoms of COVID-19 and in turn greater mortality, as the syndemic framework postulates (Albani et al., 2022; McGowan & Bambra, 2022). In terms of the Economic Complexity Index (ECI), the lower levels of economic complexity in the north of England are associated with significantly higher cases. This is perhaps because less economic complexity is associated with the kind of jobs that are less likely to be able to be done remotely, which in turn leads to higher levels of exposure, as shown in the DAG figure for cases (Mealy & Coyle, 2022). Conversely, economic complexity is not significantly associated with the COVID-19 death rate anywhere in England. The effect of our final measure of the local economic situation, the Gini coefficient, is less clear, with higher Gini coefficients having a positive effect on COVID-19 morbidity in some parts of England (e.g. the southwest). But in terms of COVID-19 morbidity, the relationship was negative and significant for all of North England, with the strongest relationships being further north. This finding contrasts with our prior assumptions detailed in the related DAG and studies that have shown a positive relationship between the Gini index and excess mortality post-pandemic in UK local authorities (Gaia & Baboukardos, 2023). The reason for such a discrepancy can be down to the fact that our analysis is localized, using MGWR, and this changes the general level findings and expectations. However, there are some studies from across Europe that showed no relationship between COVID-19 outcomes and inequality levels at small regional levels (Burlina & Rodríguez-Pose, 2023).

Higher proportions of men within a given local authority was associated with lower COVID-19 mortality, and this relationship was significant across the whole of England. The effect of the proportion of male population was strongest in the north of England. This is despite some evidence that COVID-19 that men had a higher mortality rate from COVID-19 compared to women, as shown in our DAG for mortality rate (Flor et al., 2022). However, it should be noted that local authorities with a higher proportion of male population have, on average, a significantly lower median age compared to local authorities with lower proportions of male population. Therefore, local authorities with higher proportions of male population have younger populations on average and higher proportions of male population are in turn associated with lower levels of COVID-19 mortality.

### 5.1 Local policy implications

Our findings have a couple of policy implications. Namely, as our analysis showed, deprivation was a strong predictor of COVID-19 mortalities across all localities. This finding is consistent with calls to include health in the Levelling-Up policy agenda that has aimed to reduce the spatial socioeconomic inequalities across the UK (Couper et al., 2023; Davey et al., 2022). Some even have argued that the inclusion of health in such a national policy to address geographical inequalities is a necessity now because COVID-19 has made the inequalities worse (Couper et al., 2023; Davey et al., 2022). This issue is coupled with other findings of our study that ethnic populations disproportionately suffered from the pandemic across all localities. There is evidence that structural inequalities are the reason for such suffering and the Levelling-Up policy might improve the situation for these people too (Irizar et al., 2023). Considering all these and informed by a systematic review findings, Davey et al. (2022) (Davey et al., 2022) postulated that a post-pandemic, health-oriented Levelling-Up policy to address social determinants of spatial health inequalities across England can include the following elements and interventions: (Burlina & Rodríguez-Pose, 2023) healthy-by-default and easy-to-use initiatives, e.g., subsidies for healthy food purchases or taxing alcohol and cigarette trade and purchase, to improve the conditions supportive for health-positive choices. This type of intervention can lead to healthier populations across the localities who will be less susceptible to chronic conditions like obesity and diabetes (as two of the main preconditions exacerbating COVID-19 outcomes) (Mansour et al., 2021). Long-term, multi-sector, and multi-pronged actions, e.g., housing and neighborhood interventions coupled with health education and promotion interventions in several settings (from schools to communities) and in tandem with social security and welfare policies, that are shown to be effective in reducing spatial health inequalities (Lak et al., 2021). Locally designed interventions, e.g., community-based infrastructure development according to and adapted to the local context, which are also shown to be effective in reducing spatial health inequalities (Sun et al., 2021). Targeting disadvantaged communities, e.g., women of low socioeconomic status, in all social and public health programs. Finally, (Shi et al., 2023) matching resources to local needs, e.g., allocation of NHS funds and resources proportionate to geographic need with more deprived areas receiving more resources, is another effective way of reducing spatial health inequalities. We continue this list and considering the importance of industrial policies in shaping the economic conditions of local authorities in the UK (Mealy & Coyle, 2022), we suggest that another type of policy or intervention to include in a health-oriented Levelling-Up agenda can be (McGowan & Bambra, 2022) using industrial strategies and policies, e.g., through economic complexity measures, to reshape the agglomeration of industrial infrastructure and opportunities to favor deprived areas (Zheng et al., 2020). This can change the complexity of production knowledge in the deprived areas which has been shown to be associated with more occupation opportunities, higher income, better social capital levels, and higher levels of health (Balland et al., 2022; Hidalgo, 2021). All these types of interventions can change the structural inequalities postulated by the syndemic framework as the social determinants of unequal exposure, susceptibility, and vulnerability that caused the spatial inequalities observed in the COVID-19 outcomes (Albani et al., 2022; McGowan & Bambra, 2022). Our study, to be specific, can inform these policies and interventions as it can help local policy makers to influence the factors we showed shape current (and future) pandemic outcomes locally.

### 5.2 Strengths and limitations

Although our study has some strength, especially its focus on localized investigation of relationships between COVID-19 and its determinants, there are a couple of issues that need to be addressed in future studies. Namely, local data on some chronic conditions like cancers and chronic pulmonary diseases might improve our understanding of the way these conditions, coupled with obesity and diabetes, shaped the COVID-19 profile of the localities (although our MGWR model was able to explain up to 90% of COVID-19 morbidity and mortality variance). Moreover, there needs to be local-level studies to help explain the relationships uncovered. For instance, the reasons for the strong relationship between ethnicity and COVID-19 outcomes in the southwest and north need proper local study. The same applies to diabetes rate, hospital features, and economic complexity which are more important in the east and southwest of the country. However, our study is informative for both researchers and policymakers as it shows that the localized relationships between COVID-19 and its determinants can differ from available evidence of generalized studies, and this can change the planning for public health improvement in the UK post-pandemic.

## 6 Conclusion

In this study we showed that there is a heterogeneity in the effect of demographic, health, social, and economic conditions on COVID-19 outcomes at local levels in the England. There were higher morbidity and mortality rates of COVID-19 concentrated in the northwest and southeast of England. The IMD has a significant impact on COVID-19 mortality in all local authorities, although the effect is the strongest in the south of England. Higher numbers of ethnic minorities are also associated with higher levels of COVID-19 mortality, with the strongest effect being found in the west of England. Such diverse spatial patterns were also find for age, gender, diabetes rate, economic complexity, and other determinants. The results of our study provide insights into how national and local policymakers can take account of localized factors to address spatial health inequalities and address future infectious disease pandemics.

## Funding

This research did not receive any specific grant from funding agencies in the public, commercial, or not-for-profit sectors.

## Ethical statement

There is no ethical consideration for this paper as public data is used.

## CRediT authorship contribution statement

**Esmaeil Khedmati Morasae:** Writing – review & editing, Writing – original draft, Visualization, Software, Methodology, Investigation, Formal analysis, Data curation, Conceptualization. **Daniel W. Derbyshire:** Writing – review & editing, Writing – original draft, Methodology, Conceptualization. **Payam Amini:** Writing – review & editing, Writing – original draft, Visualization, Software, Methodology, Formal analysis, Conceptualization. **Tahera Ebrahimi:** Writing – review & editing, Writing – original draft, Methodology, Formal analysis, Conceptualization.

## Declaration of competing interest

The authors declare that they have no known competing financial interests or personal relationships that could have appeared to influence the work reported in this paper.

## Figures and Tables

**Table:** 

Variable	Definition	Source/Dataset
High risk jobs (%)	Percent of active people working in COVID-19 risky jobs, i.e. percentage of people who work in the following jobs in each local authority: elementary occupations (include processing plant workers, security guards, chefs and taxi drivers) and caring, leisure and other service occupations. These people were at higher risks of COVID-19 infection and mortality.	Local authority profile 2020 (ONS).
Gini index	The Gini index measures the extent to which the distribution of income among individuals or households within an economy deviates from a perfectly equal distribution. It ranges between 0 (perfect equality) and 1 (perfect inequality).	Atlas of inequalities in UK: https://alasdairrae.github.io/atlasofinequality/
Hospital mortality	A weighted average of the ratio of the actual number of patients who died following hospitalisation at an NHS trust to the number that would be expected to die based on the average England figures, given the characteristics of the patients treated there, as of February 2020, as follows: ${Hospital\;mortality}_{i}=\sum_{y}^{n}\left(X_{y}\times W_{y}\right)$ Where: *i* is a local council, *y* is the NHS Trust where patients from the local councils i were admitted. *X* is the ratio of the actual number of patients who died following hospitalisation at an NHS trust y to the number that would be expected to die on the basis of average England figures, given the characteristics of the patients treated there. *W* is the estimated proportion of admissions of COVID-19 patients from a given local council i to a given Trust y*.	NHS. Summary Hospital-level Mortality Indicator (SHMI) - Deaths associated with hospitalisation, England, March 2019–February 2020. Table “SHMI data at trust level, Mar19-Feb20”. Available at: https://files.digital.nhs.uk/00/31F12 1/SHMI%20data%20files%2C%20 Mar19-Feb20.zip
Cancelled hospital	A weighted average of the number of urgent operations cancelled at the NHS Trusts used by a local council i as of February 2020, as follows: ${Cancelled\;hospital}_{i}=\sum_{y}^{n}\left(X_{y}\times W_{y}\right)$ Where: *i* is a local council, *y* are the NHS Trusts where patents from the local councils i were admitted. *X* is the number of urgent operations cancelled at the NHS Trust y. W is the estimated proportion of admissions of COVID-19 patients from a given local council i to a given Trust y*.	NHS. Critical Care Bed Capacity and Urgent Operations Cancelled. Table “Urgent Operations Cancelled February 2020”. Available at: MSitRep-February-2020-alHTC.xls (live.com)
Hospital business	A weighted average of the percentage of adult critical care beds occupied in the NHS Trusts used by a local council i as of February 2020, as follows: ${Hospital\;business}_{i}=\sum_{y}^{n}\left(X_{y}\times W_{y}\right)$ Where: *i* is a local council, *y* is the NHS Trust where patents from the local councils i were admitted. *X* is the percentage of adult critical care beds occupied in a given NHS Trust y. W is the estimated proportion of admissions of COVID-19 patients from a given local council i to a given Trust y*.	NHS. Critical Care Bed Capacity and Urgent Operations Cancelled. Table “Critical Care Beds February 2020”. Available at: MSitRep-February-2020-alHTC.xls (live.com)
Change in radius of gyration (Mile)	As a measure of mobility, we considered the radius of gyration for each locality. We used as the variable the change in radius of gyration pre- (March 2019 to March 2020) and post-COVID-19 (March 2020 to March 2021) (the first lockdown in UK started on 23^rd^ of March 2020 and the last (third) one finishing in March 2021). The mobility data in this study was collected from anonymous mobile phone users who opted-in to give access to their location data anonymously, through a General Data Protection Regulation (GDPR) compliant framework. In addition to anonymizing the data, the data provider applied noise to sensitive areas, such as home locations, to prevent re-identification. The datasets contain records of UK users from March 2019 to early March 2021. We analyze the radius of gyration defined as (González et al., 2008): ${RG}_{u}=\sqrt{\frac{1}{N_{u}}\sum_{i=1}^{N_{u}}{\left({\vec{r}}_{u}^{i}-\;{\vec{r}}_{u}^{cm}\right)}^{2}}$ where N represents the unique locations visited by the user u, ${\vec{r}}_{u}^{i}$ is the geographic coordinate of location I, and ${\vec{r}}_{u}^{cm}$ indicates the center of mass of the user trajectory. To put it simply, the radius of gyration defines the radius of the circle within which each users are more likely to be found. It is centered in all the visited locations by a user and is weighted by the number of times each location is visited. The data set analysed in this study contained mobility records of 1 billion de-identified, opted-in users. The data used was a weekly, monthly, and yearly time snapshot of the median radius of gyration of the users who resided in each single local authority. González MC, Hidalgo CA, Barabási AL. Understanding individual human mobility patterns. Nature. 2008 Jun 5; 453(7196):779-82. https://doi.org/10.1038/nature06958. PMID: 18528393.	Mobility data are available from Cuebiq. (www.cuebiq.com). Restrictions apply to the availability of these data. Data are available from the corresponding author with the permission of Cuebiq.
ECI 2019	We calculated the economic complexity index for UK local authorities by drawing on 3 digit industrial employment data from the Business Register and Employment Survey for the year 2019 (BRES, 2019). To calculate the ECI based on these data, we followed the approach set out in Mealy and Coyle (2021) and first construct a binary matrix M where the rows correspond to UK local authorities and the columns correspond to industries and the matrix entries are based on local authorities' location quotients in different industries (Mealy & Coyle, 2021). Location quotients are a useful way of quantifying how concentrated a particular industry is in a location relative to the national average. The location quotient for industry j in local authority i is given by: $LQ_{ij}=\left(E_{ij}/\sum_{ij}E_{ij}\right)/\left(\sum_{i}E_{ij}/\sum_{i}\sum_{j}E_{ij}\right)$ where $E_{ij}$ represents the number of people in local authority i employed in industry j. Here, a location quotient greater than 1 indicates that the local authority's employment share in that particular industry is greater than the national average. We populated the entries of the binary matrix M by letting $M_{ij}=1$ if the location quotient for industry j in local authority i is greater than 1, and $M_{ij}=0$ otherwise. A local authority's *diversity* ($d_{i}$) is defined as the number of industries that it has with a location quotient greater than 1 in (i.e. $\sum_{j}M_{i}$), while an industry's *ubiquity* ($u_{j}$) is defined as the number of local authorities that have a location quotient greater than 1 in that industry (i.e. $\sum_{i}M_{i}$). We then calculated a local authority similarity matrix given by: $\tilde{M}=D^{-1}MU^{-1}M^{\prime }$ where D and U are diagonal matrices formed respectively by local authority diversity values and industry ubiquity values along the diagonal. This $\tilde{M}$ matrix captures how similar each local authorities' industrial concentrations are to another ((Mealy et al., 2019)). Finally, we calculated the economic complexity index (ECI) for UK local authorities by finding the eigenvector associated with the second largest right eigenvalue of the $\tilde{M}$ matrix. BRES. 2019. Business Register and Employment Survey: open access [Online]. Available: Business Register and Employment Survey - Office for National Statistics (ons.gov.uk) Mealy, P., Coyle, D. To them that hath: economic complexity and local industrial strategy in the UK. Int Tax Public Finance 29, 358–377 (2022). https://doi.org/10.1007/s10797-021-09667-0 Mealy, P., Farmer, J.D. and Teytelboym, A., 2019. Interpreting economic complexity. Science advances, 5(1), p.eaau1705.	BRES. 2019. Business Register and Employment Survey: Business Register and Employment Survey - Office for National Statistics (ons.gov.uk)

*The data about the mapping on the usage of NHS Trust by local councils are based on the counts of the number of COVID-19 hospital discharges between January and September 2020 from an NHS Trust to lower-tier local authorities. Available at: https://epiforecasts.io/covid19.nhs.data/articles/mapping_summary.html.

**Figure:**
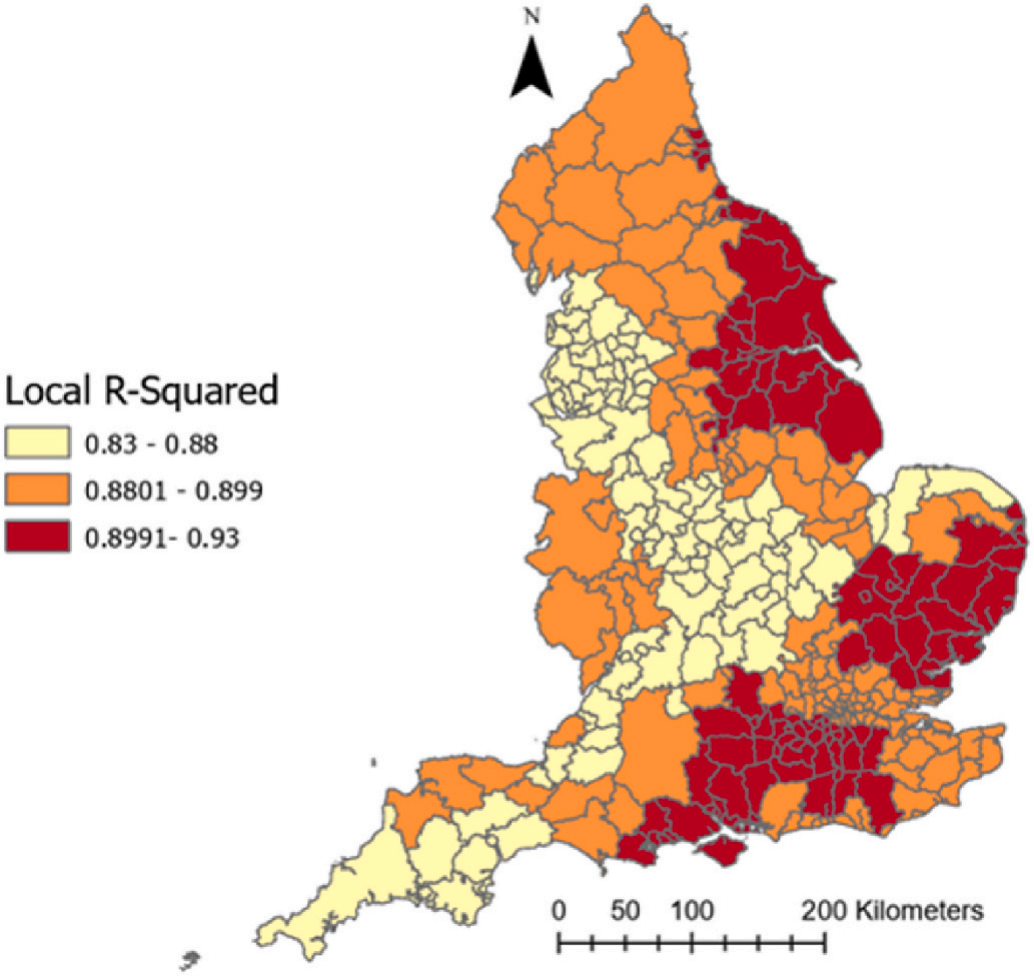


**Table:** 

Moran's index	0.2768
Expected index	−0.0030
Variance	0.0007
Z-score	32.7327
p-value	0.000

**Table:** 

Moran's index	0.2772
Expected index	−0.0030
Variance	0.0007
Z-score	32.7872
p-value	0.000

**Table:** Multi-collinearity test for cases or infection/morbidity rate:

Explanatory variables	VIF (minimum)	VIF (maximum)
Smoking over 18 (%)	0.000	0.87
Diabetes rate (%)	0.000	0.24
IMD 2019	0.000	0.97
Gini index	0.000	0.97
Obesity rate (%)	0.000	0.97
Population density (People per Sq Km)	0.000	0.64
ECI 2019	0.000	0.82
Ethnic groups (%)	0.000	0.96
Median age	0.000	0.26
Male population (%)	0.000	0.9
High risk occupations (%)	0.000	0.58
Radius of gyration change	0.000	0.38
Lockdown duration	0.000	1.00

**Table:** Multi-collinearity test for deaths or mortality rate:

Explanatory variables	VIF (minimum)	VIF (maximum)
Smoking over 18 (%)	0.000	0.4
Diabetes rate (%)	0.000	0.85
IMD 2019	0.000	0.28
Gini index	0.000	0.71
Cancelled hospital appointments (%)	0.000	0.14
Hospital business (%)	0.000	0.62
Hospital mortality (%)	0.000	0.98
Obesity rate (%)	0.000	0.95
Aged 65+ (%)	0.000	0.92
ECI 2019	0.000	0.39
Ethnic groups (%)	0.000	0.69
Male population (%)	0.000	0.87
Lockdown duration	0.000	0.99

**Figure:**
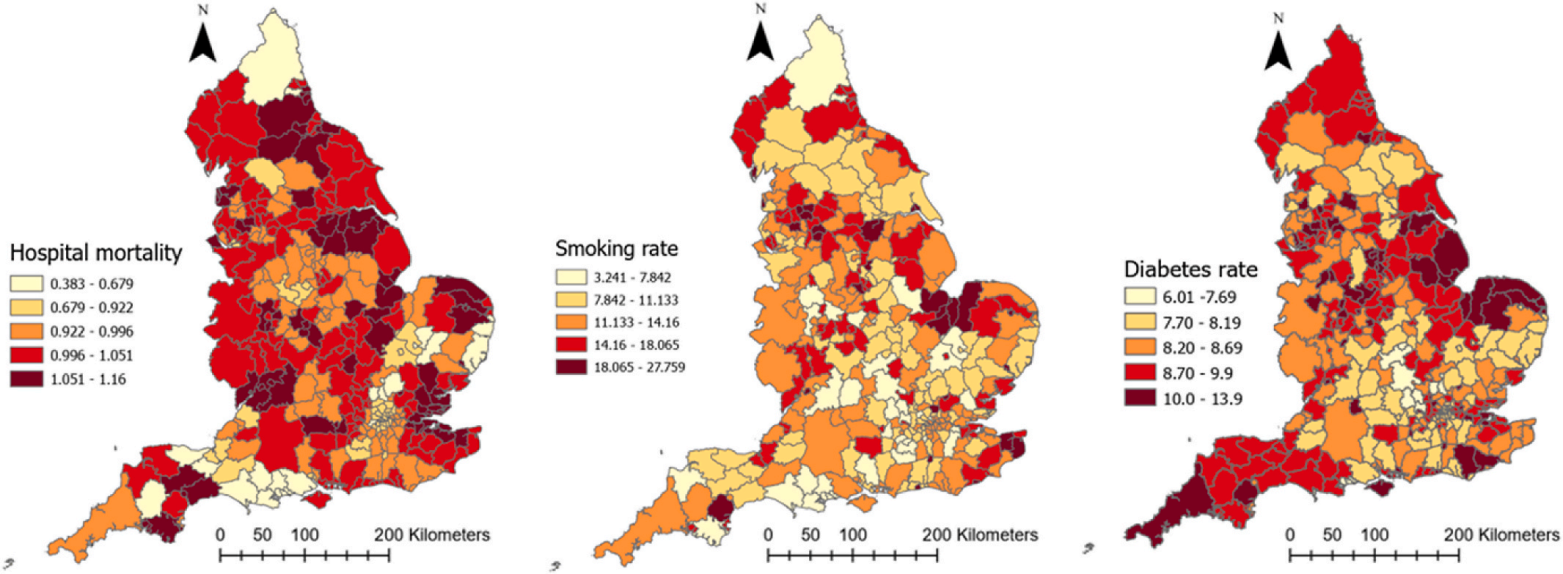


**Figure:**
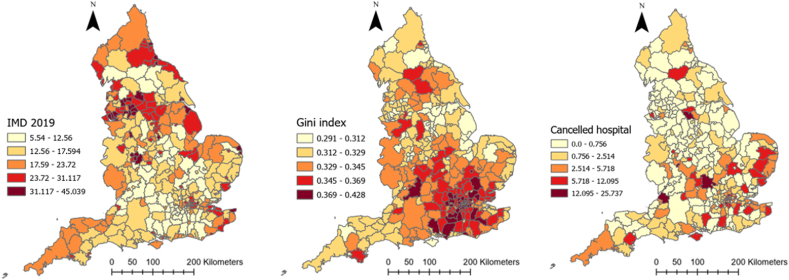


**Figure:**
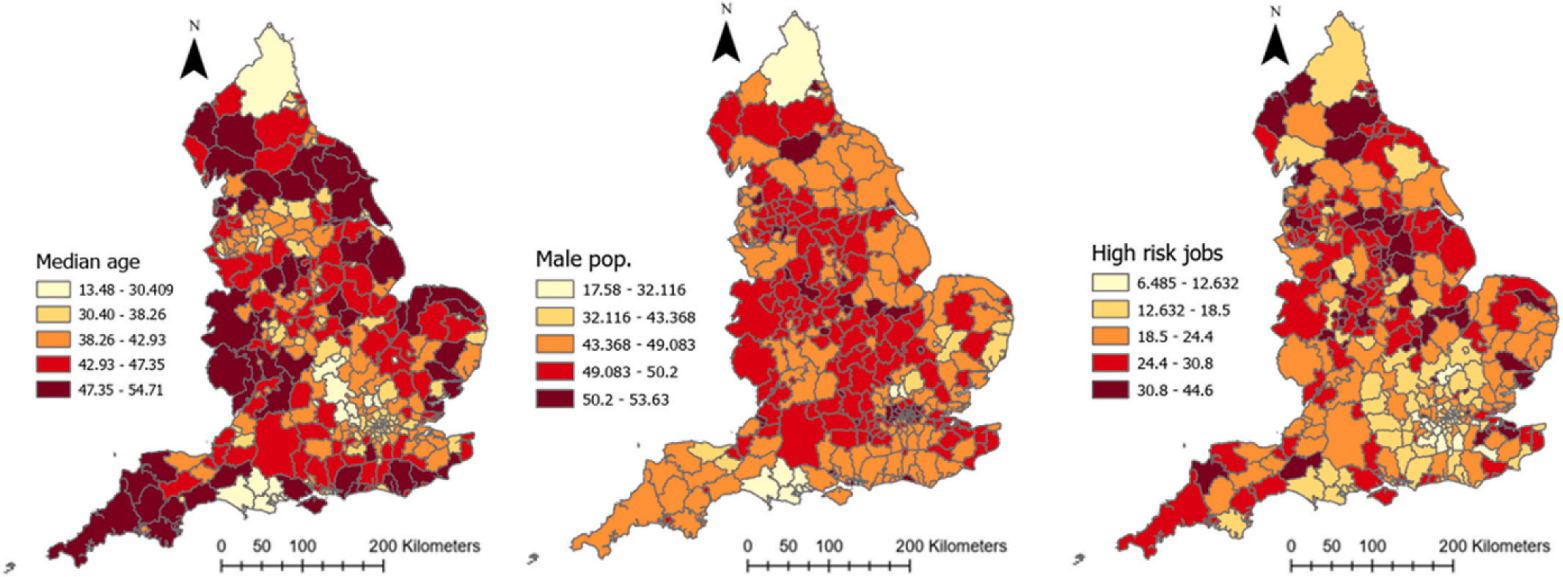


**Figure:**
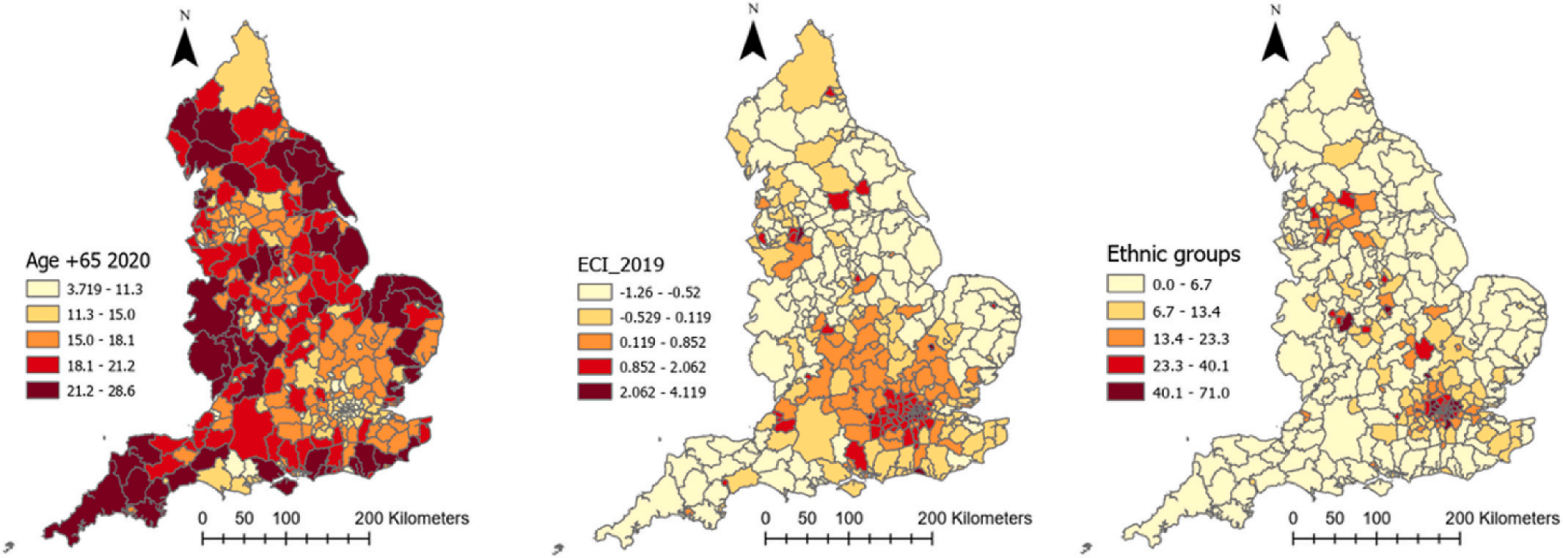


**Figure:**
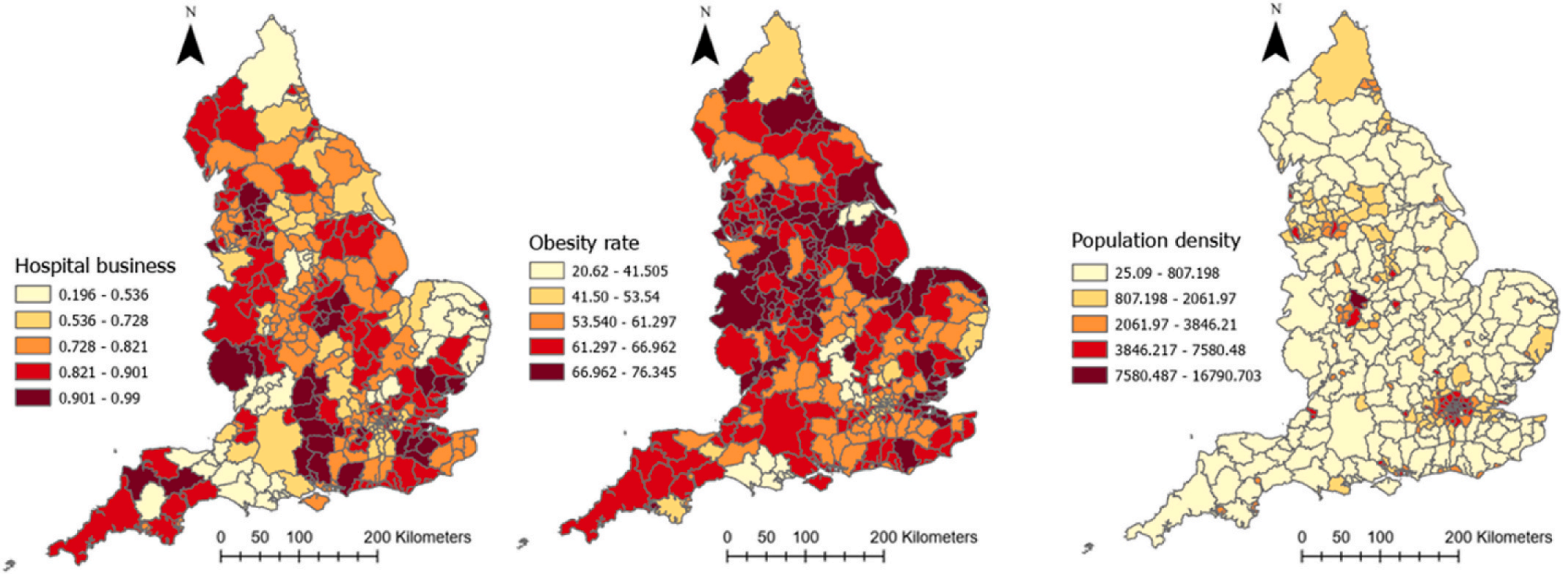


**Figure:**
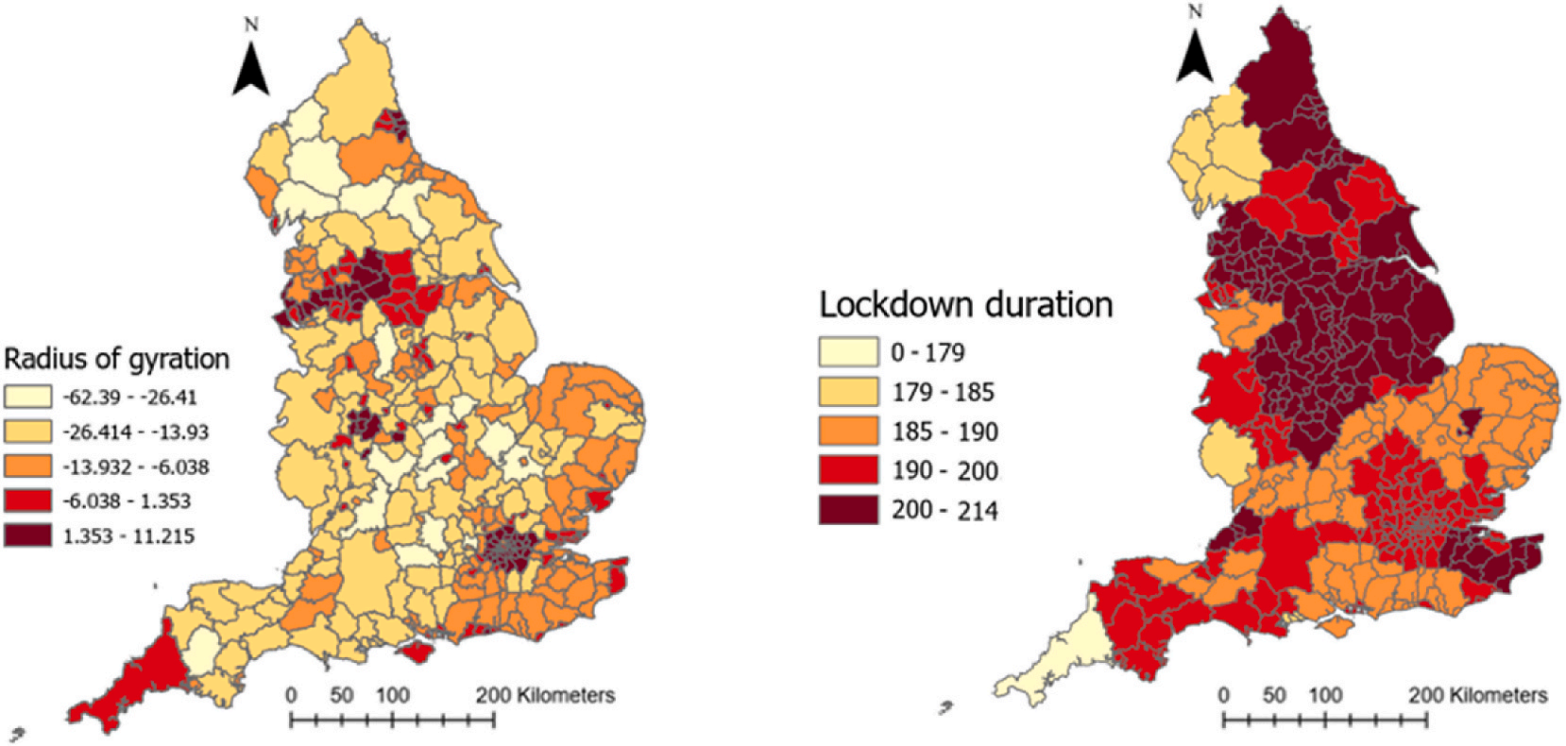


## Data Availability

Data will be made available on request.

## Appendix B

### Information related to MGWR

Local weighting scheme (Kernel function): Bisquare

Optimal bandwidth: 106 (K nearest neighbors)

Weighting method: adaptive.

Model goodness of fit (local R-squared) figure:

### Spatial autocorrelation test (Moran's test) on outcome variables:

We used Moran's test to test for autocorrelation in outcome variables. The output of Moran's test shows that there is a highly clustered pattern for both outcomes (their spatial pattern is not random), and MGWR was right approach to adopt in our study to investigate spatial features of COVID-19 in the UK. Moran's test output for each outcome variable is reported below:

Infection/morbidity rate:

• Null hypothesis: the spatial pattern observed is random.
• The P-value is 0.000, so null is rejected
• Positive Moran's index means a tendency towards clustering.
• Z score is very high, meaning the likelihood of the spatial pattern being random is extremely low.

Mortality rate:

• Null hypothesis: the spatial pattern observed is random.
• The P-value is 0.000, so null is rejected
• Positive Moran's index means a tendency towards clustering
• Z score is very high, meaning the likelihood of the spatial pattern being random is extremely low.

### Multi-collinearity test (Variance Inflation Factor (VIF)):

Local multi-collinearity is reported for each variable across the observations (local authorities). In our case, the VIF rarely passes 1, so there is no multi-collinearity between explanatory variables in this spatial scale. We report the minimum and maximum VIF value observed for each explanatory variable across the 326 local authorities.
